# Site-Directed Mutagenesis of a Hyperthermophilic Endoglucanase Cel12B from *Thermotoga maritima* Based on Rational Design

**DOI:** 10.1371/journal.pone.0133824

**Published:** 2015-07-28

**Authors:** Jinfeng Zhang, Hao Shi, Linyu Xu, Xiaoyan Zhu, Xiangqian Li

**Affiliations:** 1 Jiangsu Provincial Engineering Laboratory for Biomass Conversion and Process Integration, Huaiyin Institute of Technology, Huaian, Jiangsu 223003, P. R. China; 2 School of Life Science and Chemical Engineering, Huaiyin Institute of Technology, Huaian, Jiangsu 223003, P. R. China; 3 Jiangsu Key Laboratory for Biomass-based Energy and Enzyme Technology, Huaiyin Normal University, Huaian, Jiangsu 223300, P. R. China; Virginia Tech, UNITED STATES

## Abstract

To meet the demand for the application of high activity and thermostable cellulases in the production of new-generation bioethanol from nongrain-cellulose sources, a hyperthermostable β-1,4-endoglucase Cel12B from *Thermotoga maritima* was selected for further modification by gene site-directed mutagenesis method in the present study, based on homology modeling and rational design. As a result, two recombinant enzymes showed significant improvement in enzyme activity by 77% and 87%, respectively, higher than the parental enzyme *Tm*Cel12B. Furthermore, the two mutants could retain 80% and 90.5% of their initial activity after incubation at 80°C for 8 h, while only 45% for 5 h to *Tm*Cel12B. The *K*_m_ and *V*_max_ of the two recombinant enzymes were 1.97±0.05 mM, 4.23±0.15 μmol·mg^-1^·min^-1^ of *Tm*Cel12B-E225H-K207G-D37V, and 2.97±0.12 mM, 3.15±0.21 μmol·mg^-1^·min^-1^ of *Tm*Cel12B-E225H-K207G, respectively, when using CMC-Na as the substrate. The roles of the mutation sites were also analyzed and evaluated in terms of electron density, hydrophobicity of the modeled protein structures. The recombinant enzymes may be used in the hydrolysis of cellulose at higher temperature in the future. It was concluded that the gene mutagenesis approach of a certain active residues may effectively improve the performance of cellulases for the industrial applications and contribute to the study the thermostable mechanism of thermophilic enzymes.

## Introduction

As the ever-increasing demand for sustainable development and pressure of fossil fuel on environmental protection, there is a strong need to search for renewable energy sources, especially biomass energy [1]. The new-generation bioethanol industry utilizing renewable non-grain cellulose resources, mostly less-cost lignocellulose, is important for the sustainable development of human beings [2, 3]. Although the composition varies between different plants, 40–50% of the dry weight of biomass is comprised of cellulose, 25–30% of hemicelluloses, and the remaining made up of lignin [4]. The current bioethanol production is physically and economically constrained by high preprocessing cost and lack of suitable high effective and active cellulases, acting at high temperature and extreme pH conditions [5].

Cellulases have been widely used in the detergent, textile, and food industries, and recently for the production of bioethanol [6]. Enzymatic hydrolysis of cellulose is complicated, requiring three distinct classes of cellulases, that is endoglucanase (E.C. 3.2.1.4, also endocellulase), cellobiohydrolase (E.C. 3.2.1.91, also exocellulase), and β-glucosidase (E.C. 3.2.1.21) to function synergistically to break down the biopolymer, among which endoglucanase act by cleaving internal β-glycosidic bonds in the cellulose chain, thereby making chain ends accessible to other enzymes, therefore received relatively more attention [4, 7–9].

Due to the extreme temperature involved in the biofuel production, hyperthermophilic cellulases are considered promising for their typically valuable characteristics in the industrial utilization, and appeared to be the principal target enzymes investigated. Hyperthermophilic endoglucanases may effectively reduce the final production cost and significantly simplify the processing procedures [10–12]. Endoglucanases from extremophiles may function at higher temperatures and extreme pH changes due to their unique characteristics [8, 13, 14]. The genome of *Thermotoga maritima*, a fermentative, anaerobic, extreme thermophilic bacteria growing at high temperature up to 85°C, encodes eight endoglucanases, designated as TM0305 (Cel74), TM1048, TM1049, TM1050, TM1524 (Cel12A), TM1525 (Cel12B), TM1751 (Cel5A), and TM1752 (Cel5B), respectively, among these Cel12B is an extracellular enzymes with a signal peptide sequence at the amino terminus [15, 16]. Due to its hyperthermophilic nature, β-1,4-endoglucanase Cel12B from *T*. *maritima* (*Tm*Cel12B) may be a suitable candidate for industrial applications [17].

Directed evolution, independent of knowledge of the protein structure and the enzyme-substrate interactions, has been widely used to engineer improved mutants with enhanced activities, prolonged thermostability, and changed pH or temperature optimum, etc [10]. Rational design, a logical approach to investigate function of amino acid sites in a 3-dimensional structure of protein, requires detailed knowledge of the protein structure, and the ideally structure-function relationship. Analysis of the high-resolution three-dimensional structures of protein crystals could contribute to the deeply understanding of its biochemical function [18, 19]. If the crystal structure of one protein has not yet known, the reasonable three-dimensional structural model can be obtained by homology modelling approach using a protein structure, usually in the same family, as template whose high-resolution crystal structure has been determined at that time and possesses high sequence similarity with the target protein. The fact that an increasing number of high-resolution three-dimensional structures of endoglucases from both bacterial and fungal sources have been determined allows us to investigate the structure-function relationship [18, 20, 21].

Research on the thermostable mechanism of endoglucanase derived from extremely thermophilic source is still a hot spot [22, 23]. Rational design was carried out for improving enzyme properties of *Tm*Cel12B, such as the enhanced catalytic activity, the better thermostability and the wider substrate specificity [24–26]. An investigation of directed evolution for *Tm*Cel12B to improve enzyme activity indicated several residues playing significant roles in the catalytic hydrolysis of cellulose [27].

In the present study a systematic study was performed through constructing site-directed mutagenesis mutants of *Tm*Cel12B by the aid of rational design to obtain desirable better thermostable candidates for further study. The pH and temperature profiles of these variants were also determined to evaluate the catalytic efficiency of these mutants.

## Materials and Methods

### Strains, media and chemicals

*E*. *coli* DH5α and *E*. *coli* BL21 (DE3) (Promega Company), used as hosts for the propagation of plasmids and recombinant proteins expression, respectively, were grown in Luria-Bertani (LB) media containing yeast extract 5 g·L^-1^, tryptone 10 g·L^-1^, NaCl 5 g·L^-1^ at 37°C and pH7.0 with appropriate antibiotic supplemented. *T*. *maritima* was purchased from American Type Culture Collection (ATCC 43589). The pET-20b plasmid was obtained from Novagen Co. Ltd. Restriction enzyme, *Pyrobest* DNA polymerase, T4 polynucleotide kinase, T4 ligase, DNA marker, and *Dpn*I were purchased from Takara (Dalian, China) and used according to the manufacture’s instructions. Gene purification kit and plasmid extraction kit were purchased from QIAGEN Company. Ampicillin and isopropyl β-D-1-thiogalactopyranoside (IPTG) were obtained from Sigma-Aldrich Co. Ltd. All other chemicals used in the study were of analytical-grade purity from commercial sources unless mentioned otherwise.

### DNA manipulation and site-directed mutagenesis

The gene encoding *T*. *maritima* endoglucanase Cel12B (Gene ID: 897395, TM1525) was cloned previously by our laboratory [28]. To avoid recombinant *E*. *coli* secreting enzyme to the fermentation broth, the encoding gene of signal peptide sequence of *Tm*Cel12B was removed using two primers:

P1: 5′-GGAATTCCATATGAGGTGGGCAGTTCTTCTGA-3′,

P2: 5′-CCCAAGCTTTTATTACTCGAGTTTTACACCTTCGACAGAGAAGTC-3′,

the underlined representing *Nde*I and *Xhol* sites, respectively, then the gene without signal peptide was cloned into plasmid pET-20b digested by the regarding endonucleases to construct the recombinant plasmid for the further study of the subsequent site-directed mutagenesis. The *Tm*Cel12B-encoding gene was ligated to the pET-20b plasmid (Novagen) after digesting with *Nde I* and *Xhol*, respectively, to afford the expression vector pET-20b-*ce*l12B, carrying an N-terminal and a C-terminal 6×His-Tag sequence. Site-directed mutagenesis manipulation in DNA was performed according to the inverse polymerase chain reaction (IPCR) approach by *Pyrobest* DNA polymerase with recombinant pET-20b-*ce*l12B as the template. The sequences of the primers for different site-directed mutations, which were chosen based on the knowledge of the 3D structure of *Tm*Cel12B by the aid of homology modelling method with SWISS-MODEL tools [29–31], are listed in S1 Table. PCR reaction was designed as follows: 94°C, 5 min; 30 cycles of 94°C for 30 s, 55°C for 30 s and 72°C for 90 s; and 72°C, 10 min for extend. The PCR products were cleaned with *Dpn*I and purified with PCR purification kit (Shanghai, China), followed by phosphorylation using T4 polynucleotide kinase and ligated with T4 ligase. And finally, the recombinants were transformed into *E*. *coli* BL21 (DE3) and the positive clones were selected. Agarose gel electrophoresis was conducted according to the standard protocol [32] unless stated otherwise.

### Protein expression and purification

*E*. *coli* BL21 (DE3) cells harboring site-directed mutant of *Tm*Cel12B-encoding genes were grown in LB media containing ampicillin of 50 mg·mL^-1^ at 37°C. Production of mutated enzyme was induced by the addition of 0.5 mM IPTG when the optical density at 600 nm of the culture reached between 0.6 and 0.8, and then incubated for 5 h. The cells were harvested by centrifugation at 4°C and 10000 rpm for 10 min and washed twice with physiological saline solution (0.9% NaCl), and then resuspended in 20 mM Tris-HCl buffer (pH 7.9) containing 5 mM imidazole and 0.5 M NaCl. The cells were lysed by sonication and heat treated at 75°C for 15 min, then cooled in an ice bath. The soluble fractions of suspension were obtained by centrifugation at 10000 rpm at 4°C for 10 min. The crude enzyme extracts were retained for purification with a 1-mL of Ni^2+^-NTA affinity column (Novagen, USA) previously equilibrated with a binding buffer (20 mM potassium phosphate buffer, pH 8.0; 300 mM NaCl). Unbound proteins were washed out from the column with a washing buffer (20 mM potassium phosphate buffer, pH 8.0; 300 mM NaCl; 50 mM imidazole). The bounded proteins were eluted by a continuous gradient buffer (20 mM potassium phosphate buffer, pH 8.0; 300 mM NaCl; 50–500 mM imidazole). The proteins were analyzed with 12% sodium dodecyl sulfate polyacrylamide gel electrophoresis (SDS-PAGE).

### Homology modelling of *Tm*Cel12B and molecular docking

The 3-dimentional structure homology modelling of *Tm*Cel12B was generated using on-line automated Build Homology Models at SWISS-MODEL Workspace (http://swissmodel.expasy.org/) with the crystal structure of endoglucanase CelA from *T*. *maritima* (PDB accession code 3O7O, resolution 2.41 Å) as the template. The generated structure of protein was analyzed and verified by the Structural Analysis and Verification Server (version 4, UCLA MBI-SAVES). Structure-based sequence alignment was performed using the program ClustalX and ESPRIPT (ENDscript 2.0 and ESPript 3.0). Autodock 4.0 was used to study the interaction between *Tm*Cel12B as well as its mutants and substrate, so as to predict the spatial structure and changes herein.

### Enzyme activity and protein assays

Enzyme activities of wild-type and mutant enzymes were determined using 3,5-dinitrosalicylic acid (DNS) method with sodium carboxymethyl cellulose (CMC-Na) as soluble substrate, and regenerated amorphous cellulose (RAC) or Avicel as insoluble cellulosic substrates [33]. The assay was performed at 85°C for 10 min in a rotary shaker of 150 rpm with a 0.2 mL mixture of containing 50 mM imidazole-potassium buffer (pH 6.0), 0.5% substrate, and 50 μL of enzyme liquid. At the end of the reaction, 0.3 mL of DNS was added into the mixture and placed in a boiling water bath to stop the reaction. The amount of reducing sugars was determined by measuring the absorbance of the mixture at 540 nm. To Avicel 0.02% of 10 M NaOH was added, and alkalinized solid cellulose pellets were re-suspended into 1% SDS solution and incubated at 80°C for 15 min. One unit of enzyme activity was defined as the amount of enzyme to produce 1 μmol of reducing sugars per minute under the assay conditions described above. The concentration of protein was quantitative analysis conducted using the Bradford method with bovine serum albumin as a standard [34]. All data in the present article were replicated three times and the mean standard deviations are reported.

### Characterization of the recombinant enzymes

For analyzing the optimal reaction temperature, the activities of the recombinant enzymes were determined at different temperature ranging from 50 to 100°C. The relative activity was calculated by comparing to the highest enzyme activity (defined as 100%). The thermostability of the enzymes was investigated by incubating the enzymes at different temperatures water bath ranging from 70 to 100°C for a series of certain times in 50 mM imidazole-potassium buffer (pH 6.0) and subsequently cooled on ice for 5 min. The residual activity of the enzyme was determined under standard conditions and the activity without incubation was used as a blank and regarded as 100%.

To investigate the pH optimum and the effect of pH on the activity of enzymes, different buffers with concentrations of 50 mM at different pH range of imidazole-potassium buffer were used, to determine the activity of enzymes at 85°C. The pH stability of enzymes were investigated by incubating samples in different buffers with vary pH ranging from 6.0 to 12.0 at 30°C, and then the residual activities were determined for a certain interval at standard assay conditions. The initial activity at different pH conditions without treatment were used as a blank and regarded as 100%. All results were obtained by performing the experiments in triplicate.

## Results

### Sequence alignment and homology modelling of *Tm*Cel12B

**Fig 1:**
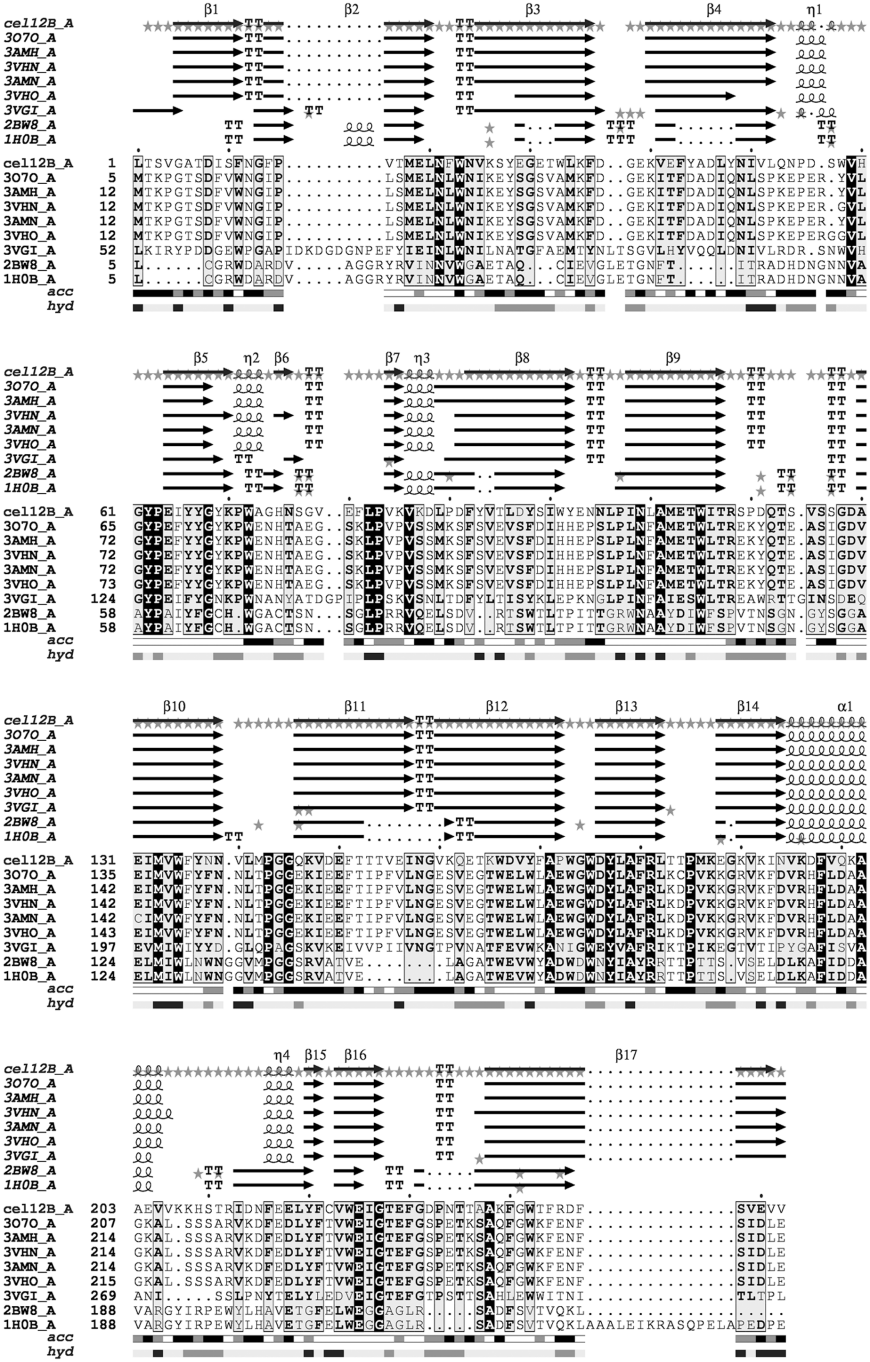
Structure-based sequence of *Tm*Cel12B and other amino acid sequences with known protein crystal structures. The secondary structure elements of the *Tm*Cel12B proteins homology modelled are drawn at the top of the alignment. The aligned protein sequences with their PDB access codes indicated in parentheses.

**Fig 2:**
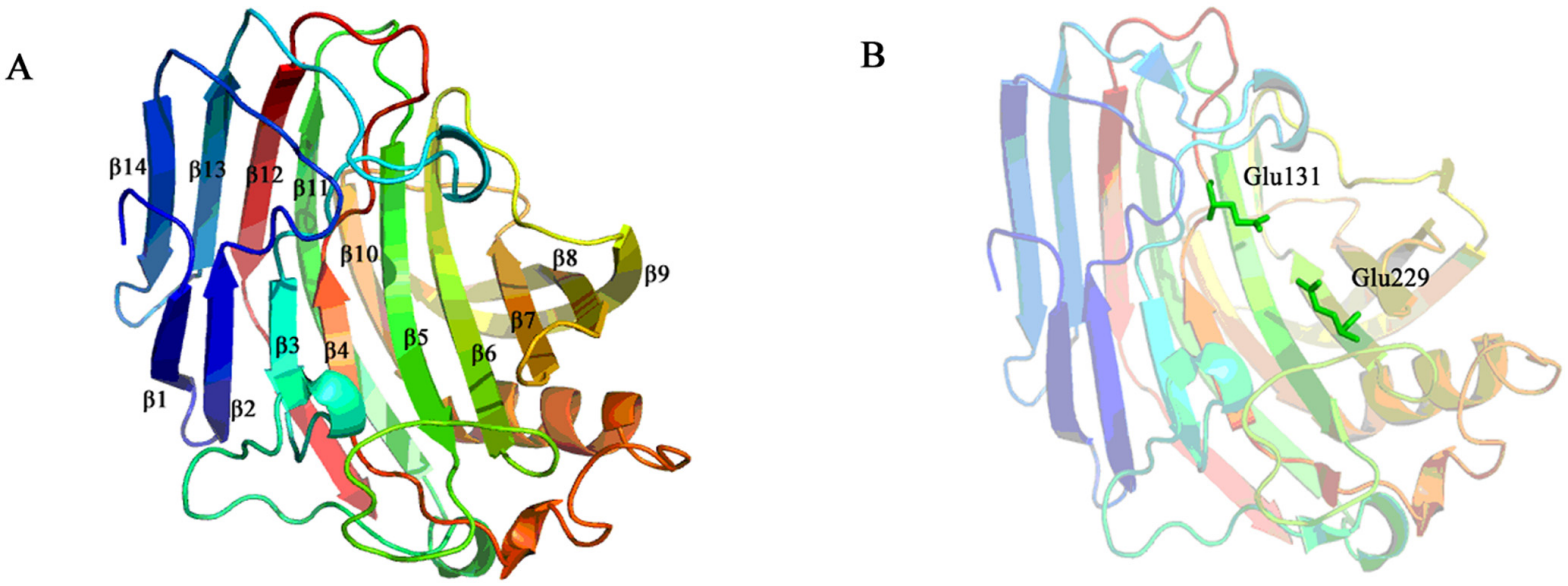
Homology model of *Tm*Cel12B. A: The secondary structural elements and loops are spectrum-colored according to their positions in the amino acid sequence. The 14 β-strands are pack against each other into two anti-parallel sheets. B: Active site Glu131 and Glu229 was colored green.

**Fig 3:**
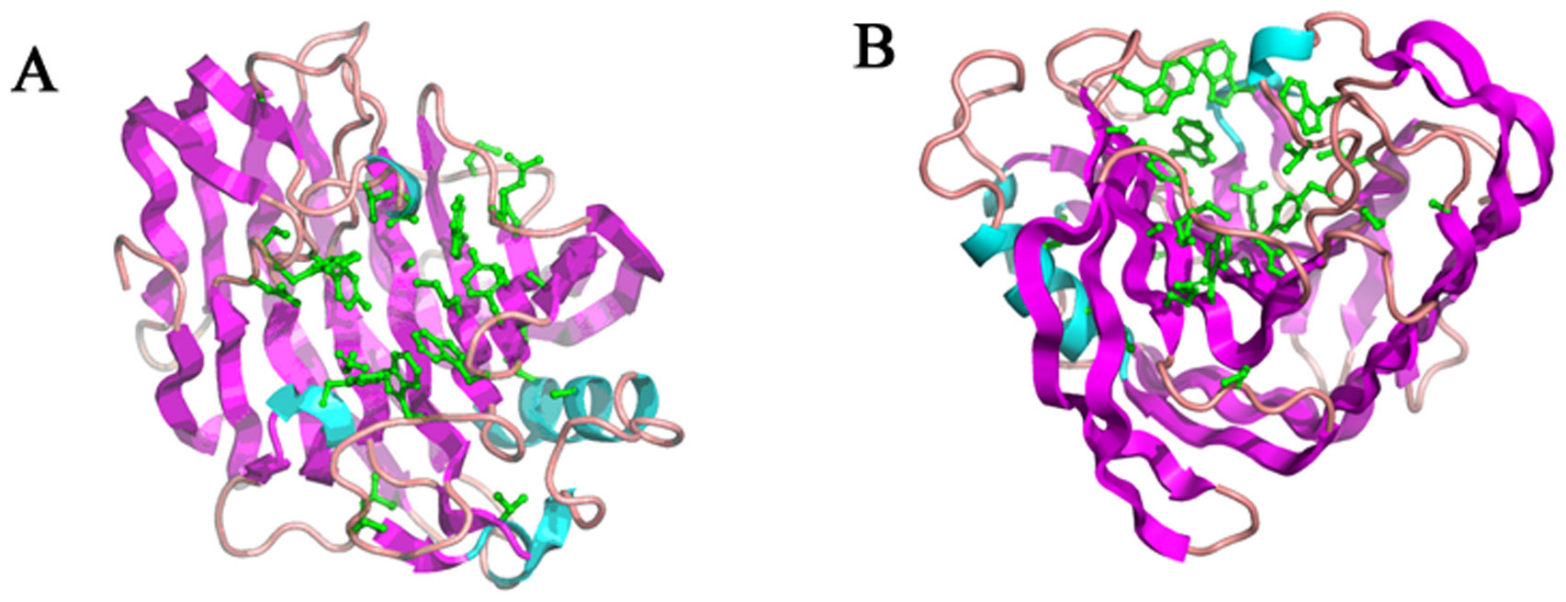
Conserved amino acid residues of thermostable endoglucanase. The conserved amino acid residues with green color are mostly located in the active cleft. A: front view. B: side view.

Different from other intracellular endoglucanase, such as Cel12A and Cel74, *Tm*Cel12B carries a typical signal peptide sequence within a hydrophobic amino-terminal extension, therefore belongs to extracellular enzymes. The sequence alignment result showed that *Tm*Cel12B has the highest similarity with the protein CelA from *T*. *maritima* (PDB accession code: 3O7O), Cel12A from *Pyrococcus furiosus* (PDB accession code: 3VGI), and Cel12A from *Rhodothermus marinus* (PDB accession code: 1H0B), which also belong to the GH12 family (Fig 1). The 3-dimensional homology models of *Tm*Cel12B and its mutants were generated by on-line SWISS-MODEL tool using a well characterized protein crystal of endoglucanase CelA from *T*. *maritima* as a model template [35]. The most probable structure of *Tm*Cel12B protein could be generated by comparative modelling with the template sequence, simultaneously satisfying spatial restraints and local molecular geometry. The modeled structure was refined by subsequent optimization of the loop conformations to assess the compatibility of the primary sequence to the template structure. The geometry of loop regions was corrected using on-line Refine Loop Program. As shown in Fig 2A, the overall fold of *Tm*Cel12B is a β-barrel jelly-roll fold structure consisting of 14 β-sheets, similar to those other enzymes from GH12 family. The two curved β-sheets folds are packed against each other, and consist of five strands and nine strands, respectively. The catalytic center is located at the bottom of the inner hole formed by the inner sheet structure, whereas the outer sheet structures will play key roles in stabilizing the whole space frame. The two catalytic residues Glu131 and Glu229, which are seated in the inner loop between sheets of β4 and β12, are embedded themselves in the catalytic cleft (Fig 2B). The first three loops of the inner sheet form a cleft covering the catalytic center hole, and allow the substrate to access to the binding module of the enzyme. Consistent with the other GH12 family endoglucanases, the interior of the protein is filled most by hydrophobic residues that constitute the protein structure core, mostly including some aromatic amino acid residues, such as phenylalanine. The central hole is also formed by several side chains of tryptophan and tyrosine serving as different roles to interact with the substrate. From Fig 3, the conserved amino acid residues within the thermostable endoglucanases with high homology were mostly located in the inner of the catalytic center cleft, which can be inferred that these conserved residues are not only responsible for the stabilization of the protein structure, but also the construction of the catalytic center.

### Site-directed mutagenesis of the functional residues

**Fig 4:**
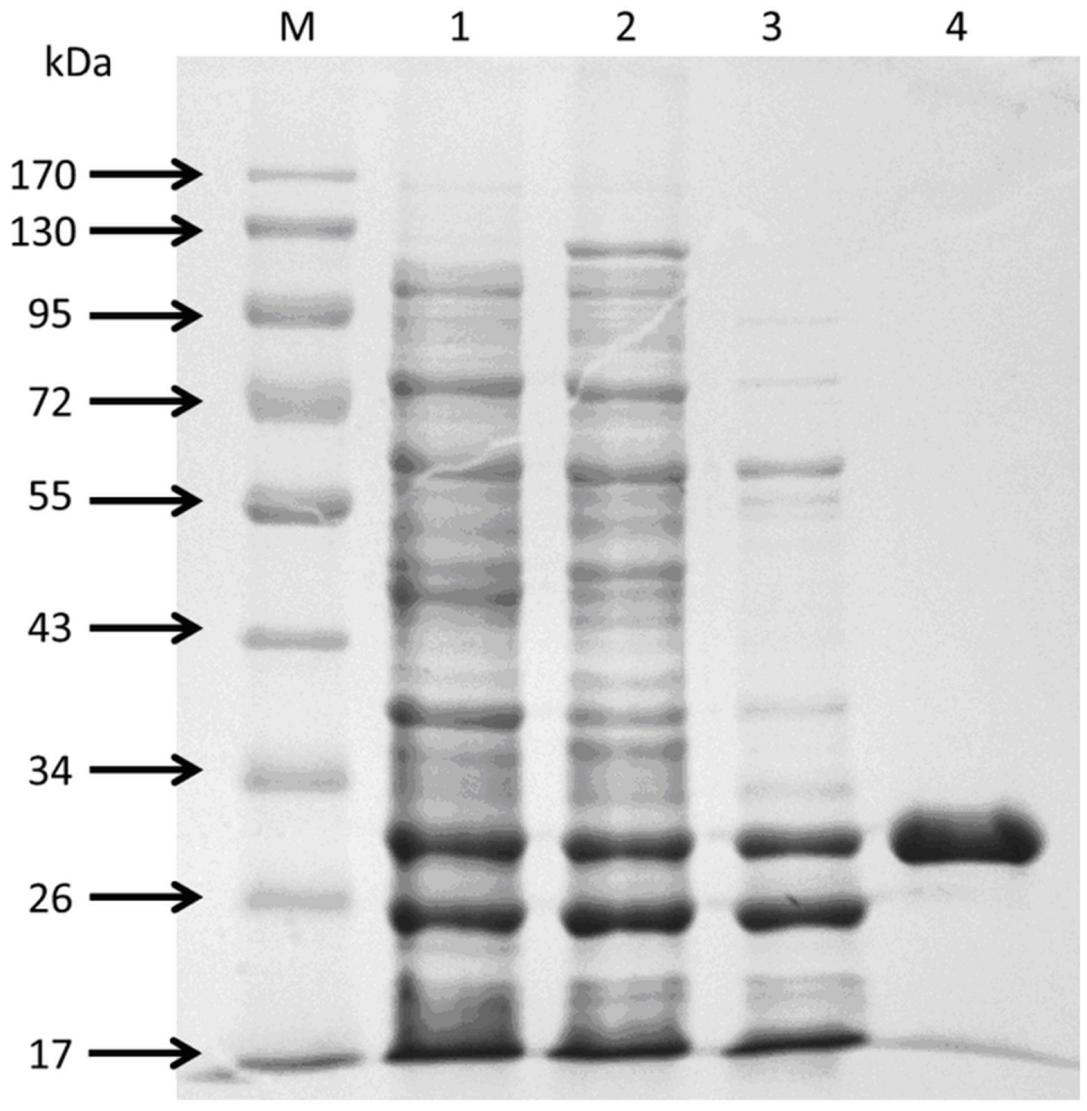
Structures of site-directed amino acid residues with other vicinal residues by H-bond. A, B: Glutamic acid 225 was mutated into Histidine. C, D: Aspartic acid 37 was mutated into Valine. E, F: Lysine 207 was mutated into Glutamic acid.

**Fig 5:**
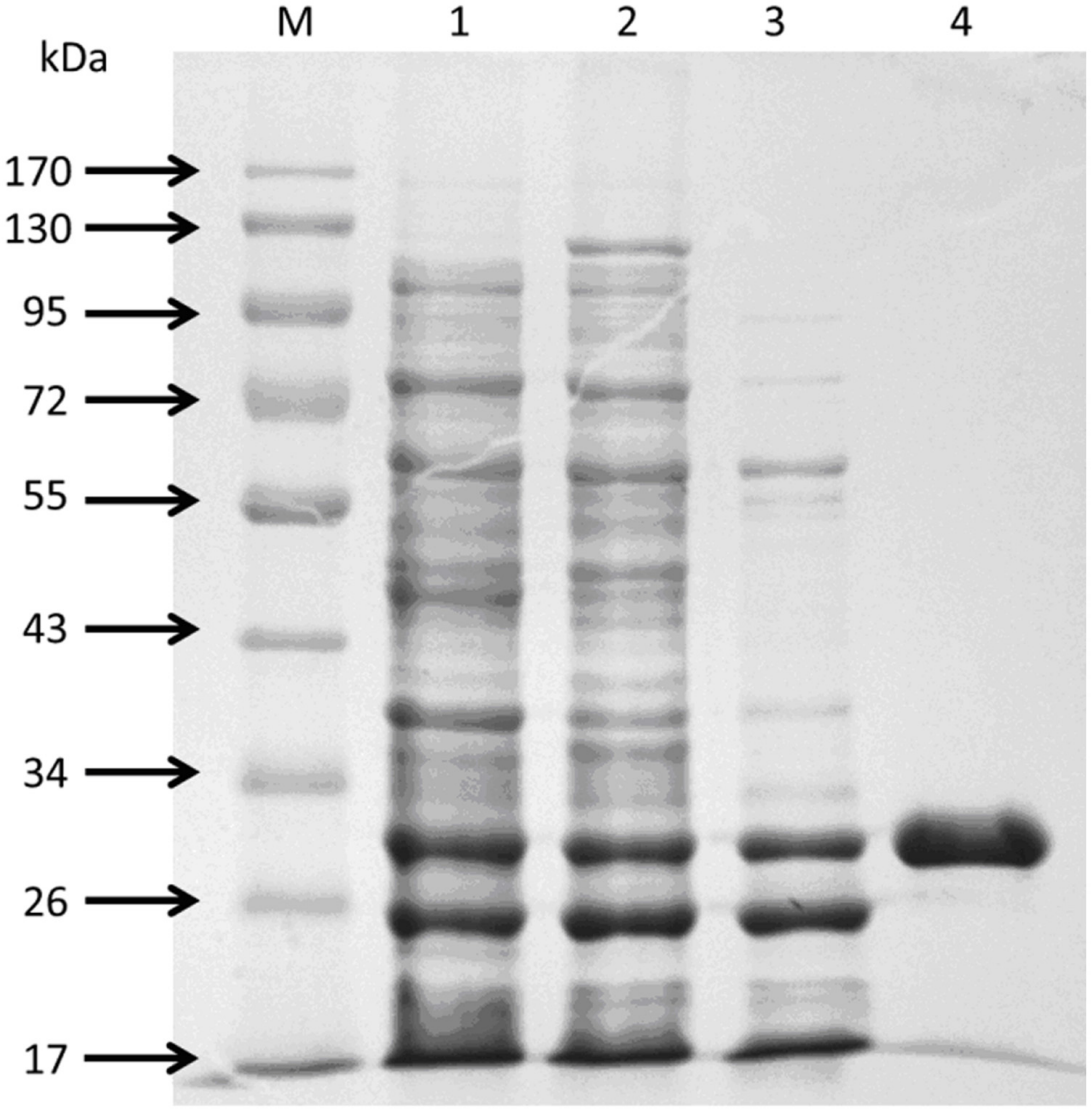
SDS-PAGE analysis of Recombinant *Tm*Cel12B. Lane M, Protein marker; Lane 1, Supernatant after ultrosonication; Lane 2, suspensions after ammonium sulfate precipitation; Lane 3, treated by heat at 75°C for 15 min; Lane 4, purified enzyme after purification kit.

A set of experiments of directed evolution using error-prone PCR approach for improving properties of *Tm*Cel12B were previously performed by our research group [27]. As the result of the directed evolution studies, five well-evolved mutants of *Tm*Cel12B with highly improved properties in terms of enzyme activity, thermostability, and pH stability, respectively, were obtained, among which the best-evolved mutant (L20R, D37V, I108T) showed about 3 times higher in the enzyme activity than its parental clone, furthermore, the substitution of amino acid residues in the primary structure, compared to the wild-type *Tm*Cel12B, were affirmed by gene-encoding sequencing. The enzymatic characteristics studies and structural analysis demonstrated that the polarity of catalytic active center was increased, as well as the enzyme structure was increased compact to a certain extent [27]. By sequencing primary structure of 5 mutants, all having 3–6 amino acid sites substitution, respectively, they share 2 common sites substitution, E225 and K207. 3 out of 5 mutants share another common substitution, D37. The results indicate that the three residues play a modest role on thermostable properties of *Tm*Cel12B. Homology modeling indicated that residue site of E225 is near the catalytic center and within the cavity of substrates accessory to the enzyme protein, while amino acid sites of K207 and D37 are distributed on both sides of the outer surface of protein molecule. Combined with the results of previous directed evolution study, a series of systematic studies were designed by the rational design strategies for the deeply analysis of the structure-function relationship. Target amino acid residues of E225, K207, and D37 were selected to perform single and combinatorial mutagenesis using site-directed reverse PCR approaches. Taking account into the polarity and hydrophobicity, the three residues were mutated into different amino acids in order to investigate the structure-function relationship. The comparison study on the residues exchange was also performed. The results can be seen in Fig 4. The interaction of the three residues with vicinal amino acids was decreased, and probably attributed to the reconstruction of the catalytic domain and substrate binding sites. The resultant recombinant mutants of *Tm*Cel12B all have apparent molecular mass of about 30 kDa determined by SDS-PAGE analysis (Fig 5), corresponding well with the desired value deduced from the nucleotide sequence of the gene.

### Enzyme activities of *Tm*Cel12B and its mutants

Compare to other thermophilic cellulases, *Tm*Cel12B possess unique hyperthermostable property but relatively low activity. Another main aim of the study is, except for enhanced thermostable, to improve the enzyme activity under high temperature conditions. Thus, the enzyme activity of endoglucanase *Tm*Cel12B and its site-directed mutants were determined under the standard conditions using CMC, RAC, and Avicel as the substrates. The results show that the two single-site mutants both show improved enzyme activities of 44% and 43% higher than their parental enzyme, respectively (S2 Table). Furthermore, the double-sites and triple-sites mutants were also significantly enhanced on catalytic performance about 77% and 87%, respectively. As to the RAC and Avicel substrates, *Tm*Cel12B and its mutants hardly show any detected hydrolysis activities.

### Temperature and pH profiles of *Tm*Cel12B and its mutants

Enzymes derived from extremely thermophilic microorganisms have also been studied for their special properties, which could be readily utilized in various industrial processes involving high temperature and strong basic or acid conditions. To evaluate the optimal temperature and pH profiles, *Tm*Cel12B and its mutants were analyzed in terms of catalytic activities at various temperatures ranging from 50°C to 100°C, or pH ranging from 4 to 10 (S2 Table). *Tm*Cel12B showed pH optimum of 6.0 and temperature optimum of 85°C against the substrate CMC-Na, which demonstrated its unique hyperthermostability. When glutamate225 was substitute for histidine, the pH optimum was slightly shifted toward 6.5, while the optimal temperature was 80°C, a little decline about 5°C compare to that of *Tm*Cel12B, though *Tm*Cel12B-E225H showed higher catalytic activity. On the other hand, the thermostability of *Tm*Cel12B-E225H showed an improvement of remaining 95% of its initial activity after incubation for 8 h at 75°C, while only 45% for *Tm*Cel12B when incubation for 2 h at 75°C. In contrast, *Tm*Cel12B-K207G showed less thermostable of 65% residual activity at 75°C for 3 h compare to that of *Tm*Cel12B-E225H. However, the pH and temperature optimum of *Tm*Cel12B-K207G appeared to be 7.5 and 95°C, respectively, higher than those of *Tm*Cel12B-E225H and *Tm*Cel12B. To obtain desired high activity recombinant enzyme, a combination of two residues mutagenesis was subsequently performed. The results indicated that the double-sites mutant *Tm*Cel12B-E225H-K207G showed pH optimum of 7.5, temperature optimum of 95°C, respectively, and better thermostability of 90.5% residual activity after incubation at 80°C for 8 h, demonstrating that the two amino acid sites were indeed account for the pH and thermostability characteristics’. Furthermore, no interaction was observed when two residues were mutated simultaneously. The triple-sites mutant *Tm*Cel12B-E225H-K207G-D37V was also constructed to investigate the role of aspartic acid site on pH and temperature profiles, as well as activity of *Tm*Cel12B. As the result, no obvious change was observed, but a little reduction on pH optimum from 7.5 to 7.0.

### Kinetic parameters

Kinetic parameters for wild-type *Tm*Cel12B and its site-directed mutants were determined, based on the initial reaction rates (ν_0_) evaluated during the first 3 min. The results can be seen in S2 Table. Though the activities of the mutated enzymes improved to varying degrees, the Michaelis constants varied in different site-directed mutants. The apparent *K*_m_ of the two single-site mutants and double-sites mutants showed an increase to a certain extent, while the triple-sites mutant appeared to be about 12% reduction. Since higher apparent *K*_m_ represents lower affinity to the substrates, higher substrate concentration is needed to afford an equal enzyme activity to those possessing lower apparent *K*_m_. Structural and conformational changes in the mutated protein structure may cause effects to a certain extent on substrates entering the catalytic active center of the enzyme and binding to the side chain of amino acid residues. The *V*_max_ and *K*_m_ of *Tm*Cel12B-E225H-K207G were 3.15±0.21 μmol·mg^-1^·min^-1^ and 2.97±0.12 mM, respectively. The *V*_max_ and *K*_m_ of the triple-sites mutant *Tm*Cel12B-E225H-K207G-D37V were 4.23±0.15 μmol·mg^-1^·min^-1^ and 1.97±0.05 mM, about two times higher than that of the initial enzyme.

## Discussions

Endoglucanases catalyzing the hydrolysis of internal β-1,4-glucasidic bonds of cellulose via a double displacement reaction and a glycosyl-enzyme intermediate, mostly belong to the members of glycoside hydrolyses (GH) family 5, 10, 12, 16, 18, 19, 26, 44, 45, 48, 51, 74, and 124 [7, 36]. GH12 family, as well as GH11 family of xylanase, constitutes Clan C of glycoside hydrolyses according to the classification by CAZy database (the Carbohydrate-Active enzymes database, http://www.cazy.org). It has been reported that two Glutamate residues of GH12 family members were approved to be essential for the catalytic activity of these enzymes [37]. *Tm*Cel12B also has these two Glutamate residue sites at the corresponding positions (E131 and E229, Fig 2B) to indicate that these two acidic residues are indeed the essential active sites of GH12 family members [17]. As to the amino acid compositions of hyperthermostable cellulases, there have been revealed some differences, compared with the less thermostable cellulases of the same family, for example generally more Glutamate residue while less Proline residue [17]. However, there are not yet enough evidences to elucidate if the compositions of primary structure are account for the extreme thermostable properties of these enzymes so far.

Since thermostability is an important and complex property, many efforts to enhance the thermostability of existing endoglucanases in question have been made, including increase hydrophobicity within catalytic center, change polarity and charge of amino acid residues in primary structure, enhance the compactness of the holoenzymate protein, etc [38, 39]. Numerous studies regarding directed evolution of cellulase have been reported to search for desirable mutants with higher thermostability and enzyme activity. As the result, many improved mutants were obtained, and different mechanisms were presented to explain the thermostable profiles of enzymes [40, 41]. There have been abundant structural reports of proteins about endoglucanases, which demonstrated three different folds present, namely (α/β)_8_, β-jelly roll, and (α/α)_6_ fold [36]. Analysis of the structure-function relationship between hyperthermostable endoglucanases showed that the type of fold is critical for distinct thermostable features. The 3-dimentional structure model of *Tm*Cel12B revealed a β-sandwich fold, comprise of two β-sheets fold packed against each other. Homology modelling study indicated that the diameter of the holes to the catalytic cavity became bigger and the structure of the protein was more compact. From Fig 4, Histidine residue appeared an active basic amino acid, usually acting as the proton donor and acceptor, thus Glu225 was mutated into Histidine to attempt to enhance the electron density of the catalytic active center, which contributed to the improvement of the enzyme activity of the mutant. The Lys207 and Asp37 residues are located in different loops of the protein structure, and now they were substituted by Glysine and Valine to increase the hydrophobicity of the outer surface of protein to some extent, so as to form a more compact enzyme complex with the substrate under the aqueous conditions. These results are supposed to be responsible not only for the enhancement on enzyme activity and thermostability, but also contribute to investigate the difference between mesophilic and thermophilic cellulases.

Rational design is now widely used to investigate for improved enzyme properties, based on the scientific knowledge of the structure-function relationship of enzymes [3]. Numerous studies using site-directed mutagenesis or directed evolution approaches have been made to investigate catalytic mechanism and improved properties [6, 42]. However, few reports obtained significantly improved mutants with higher activity on insoluble substrates [43], and so did the current study. Another important point to note is that to break down the cellulose polymer to produce bioethanol needs many cellulolytic enzymes, such as cellobiohydrolase, ligninase, β-glucasidase and xylanase, etc, to function synergistically, which is a more complex process, especially on insoluble heterogeneous cellulose, that is to say, a series of cellulases all need to modify by various approaches to form a ternary complex with the substrate. Some efforts have been made through cellulase diffusion, adsorption, and catalysis on the surface of cellulose, and construction of recombinant enzymes by combining the cellulose-binding modules (CBM) to the catalytic domain from varied source [44]. Therefore, a significant reduction in cost is crucial for industrial use in biorefinery and biofuel to meet the demand of environmental sustainable development and energy economics change.

## Conclusions

The unique hyperthermostability profiles of endoglucanase *Tm*Cel12B make it of great value in the study of biofuel production from nonfood lignocellulosic biomass. The mutants engineered in the current study are promising candidates in the recent future for cellulose hydrolysis. The study also demonstrated the feasibility of engineering cellulases in the industrial utilization, as well as provided the experimental proof of thermostable mechanism of endoglucanases based on the analysis of three-dimension protein structure.

## Supporting Information

S1 Table Primers used in site-directed mutagenesis with IPCR for the generation of mutants. (DOC)

S2 Table Characterization of *Tm*Cel12B and its mutants. (DOC)
