# Correction: Site-Directed Mutagenesis of a Hyperthermophilic Endoglucanase Cel12B from *Thermotoga maritima* Based on Rational Design

**DOI:** 10.1371/journal.pone.0141937

**Published:** 2015-10-28

**Authors:** Jinfeng Zhang, Hao Shi, Linyu Xu, Xiaoyan Zhu, Xiangqian Li

Fig 4 is incorrect. Fig 4 incorrectly appears a duplicate of Fig 5. The authors have provided a corrected version of Fig 4 here.

**Fig 4:**
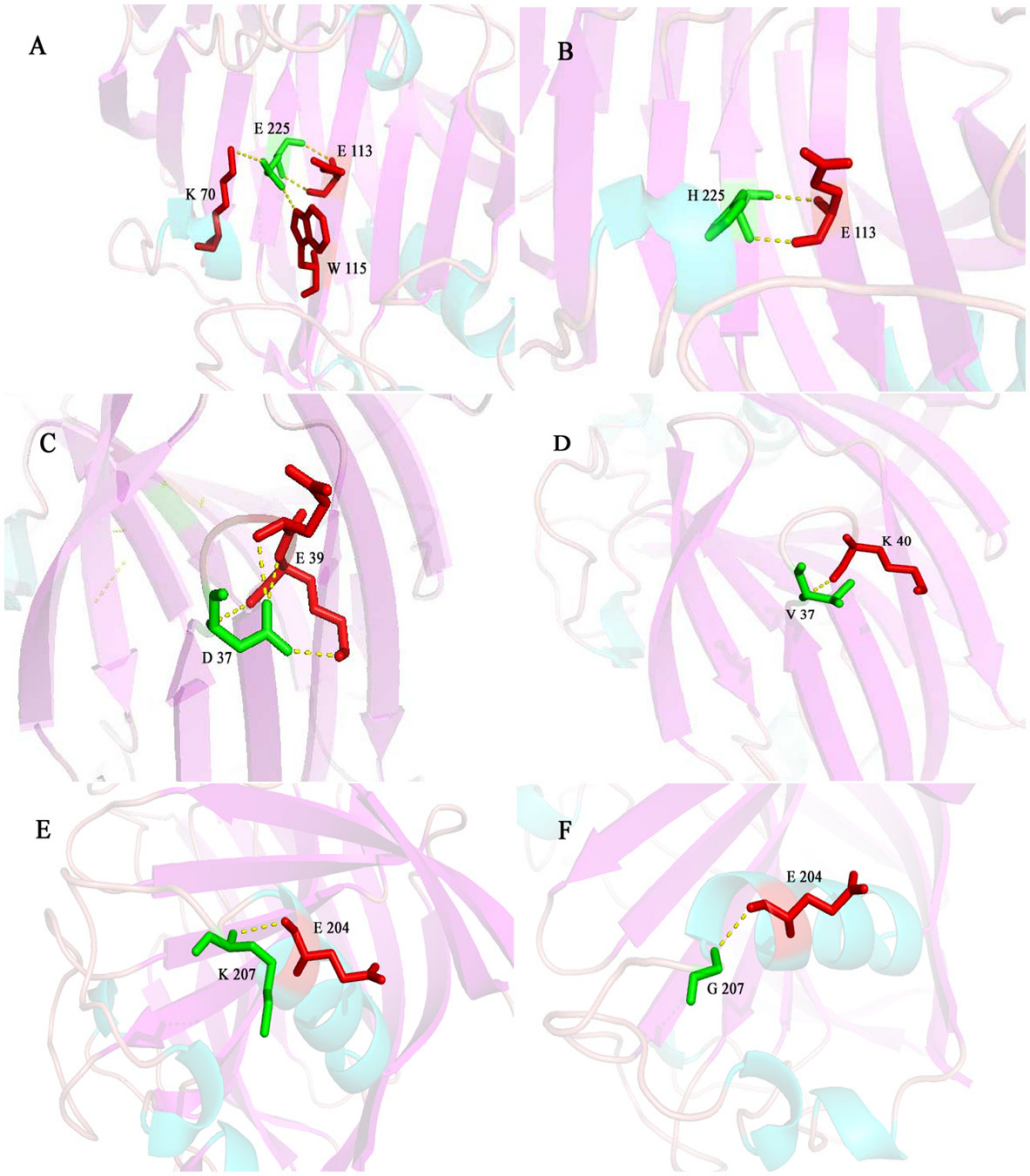
Structures of site-directed amino acid residues with other vicinal residues by H-bond. A, B: Glutamic acid 225 was mutated into Histidine. C, D: Aspartic acid 37 was mutated into Valine. E, F: Lysine 207 was mutated into Glutamic acid.
