# Supplementary material for: Site-Directed Mutagenesis of a Hyperthermophilic Endoglucanase Cel12B from Thermotoga maritima Based on Rational Design
Source: PLoS One. 2015 Jul 28;10(7):e0133824. doi: 10.1371/journal.pone.0133824 (PMC4517919; doi:10.1371/journal.pone.0133824)
Supplement: S1 Table — (DOC) [file pone.0133824.s001.doc]

Supporting Information Table 1 Primers used in site-directed mutagenesis with IPCR for the generation of mutants

| Mutation | Primer sequence |
| --- | --- |
| Glu225His  (*Tm*Cel12B-E225H) | 5'-CTGCGTCTGGCATATCGGAACGGA-3' |
|  | 5'-ATACAGCTCTTCGAAATTGTCTA-3' |
| Lys207Gly  (*Tm*Cel12B-K207G) | 5'-CGGAAGTTGTCGGTAAGCAC-3' |
|  | 5'-CGGCTTTCTGAACGAAGTCCT-3' |
| Glu225His & Lys207Gly (*Tm*Cel12B-E225H-K207G) | 5'-CGGAAGTTGTCGGTAAGCAC-3' |
|  | 5'-CGGCTTTCTGAACGAAGTCCT-3' |
| Glu225His, Lys207Gly & Asp37Val  (*Tm*Cel12B-E225H-K207G-D37V) | 5'-GCTCAAATTTGTTGGAGAAAAGGTT-3' |
|  | 5'-CACGTTTCTCCTTCATAGGATTT-3' |
