# Supplementary material for: Site-Directed Mutagenesis of a Hyperthermophilic Endoglucanase Cel12B from Thermotoga maritima Based on Rational Design
Source: PLoS One. 2015 Jul 28;10(7):e0133824. doi: 10.1371/journal.pone.0133824 (PMC4517919; doi:10.1371/journal.pone.0133824)
Supplement: S2 Table — (DOC) [file pone.0133824.s002.doc]

| Enzyme | pH_opt_ | T_opt_ (°C) | Enzyme activity (U/mL) | | | *V*_max_ (μmol·mg^-1^·min^-1^ ) | *K*_m_  (mM) |
| --- | --- | --- | --- | --- | --- | --- | --- |
|  |  |  | CMC | RAC | Avicel |  |  |
| *Tm*Cel12B | 6.0 | 85 | 2.34±0.11 | 0.53±0.09 | 0.05±0.01 | 2.05±0.32 | 6.0 |
| *Tm*Cel12B-E225H | 6.5 | 80 | 3.39±0.07 | 0.58±0.11 | 0.04±0.01 | 3.08±0.09 | 6.5 |
| *Tm*Cel12B-K207G | 7.5 | 95 | 3.37±0.09 | 0.49±0.04 | 0.05±0.01 | 2.54±0.03 | 7.5 |
| *Tm*Cel12B-E225H-K207G | 7.5 | 95 | 4.17±0.12 | 0.65±0.06 | 0.05±0.01 | 3.15±0.21 | 7.5 |
| *Tm*Cel12B-E225H-K207G-D37V | 7.0 | 95 | 4.37±0.04 | 0.60±0.10 | 0.06±0.02 | 4.23±0.15 | 7.0 |

Supporting Information Table 2 Characterization of *Tm*Cel12B and its mutants
